# Sucrase-Isomaltase Deficiency Causing Persistent Bloating and Diarrhea in an Adult Female

**DOI:** 10.7759/cureus.14349

**Published:** 2021-04-07

**Authors:** Varsha Chiruvella, Ayesha Cheema, Hafiz Muhammad Sharjeel Arshad, Jacqueline T Chan, John Erikson L Yap

**Affiliations:** 1 Internal Medicine, Medical College of Georgia at Augusta University, Augusta, USA; 2 Gastroenterology and Hepatology, Medical College of Georgia at Augusta University, Augusta, USA; 3 Pediatric Endocrinology, Medical College of Georgia at Augusta University, Augusta, USA

**Keywords:** sucrase-isomaltase, starch intolerance, disaccharidase assay, ibs, hydrogen breath test

## Abstract

Congenital sucrase isomaltase deficiency (CSID) is an autosomal recessive disorder which leads to chronic intestinal malabsorption of nutrients from ingested starch and sucrose. Symptoms usually present after consumption of fruits, juices, grains, and starches, leading to failure to thrive and malnutrition. Diagnosis is suspected on detailed patient history and confirmed by a disaccharidase assay using small intestinal biopsies or sucrose hydrogen breath test. Treatment of CSID consists of limiting sucrose in diet and replacement therapy with sacrosidase. Due to its nonspecific symptoms, CSID may be undiagnosed in many patients for several years. We present a case of a 50-year-old woman with persistent symptoms of bloating in spite of extensive evaluation and treatment.

## Introduction

Congenital sucrase isomaltase deficiency (CSID), also known as genetic sucrase deficiency, is a multifaceted intestinal malabsorption disorder with an autosomal recessive mutation in the sucrase-isomaltase (SI) gene on chromosome 3 (3q25-q26). Sucrase-isomaltase is a type II membrane enzyme complex and member of the disaccharidase family required for the breakdown of α-glycosidic linkages in sucrose and maltose. When this enzyme complex is deficient, nutrients from ingested starch and sucrose cannot be absorbed sufficiently. Infants and young children generally develop symptoms such as explosive watery diarrhea, abdominal distension, colic, and dehydration after consumption of fruits, juices, grains, modified milk formulas, and starches. These dietary symptoms reduce an individual's absorption of essential dietary nutrients and cause malnutrition. In infants and children, this commonly manifests as failure to gain appropriate weight and height and presents as low body weight in adults. However, starch intolerance often disappears during childhood, and symptoms of sucrose intolerance usually improve as the affected child ages [1]. A confirmatory CSID diagnosis can be made by a disaccharidase assay using a small bowel tissue biopsy or sucrose breath testing. Treatment for the condition consists of a combination approach of diet modification limiting sucrose and sucrase enzyme replacement therapy with sacrosidase, obtained from baker’s yeast and glycerin, which is the only Food and Drug Administration (FDA) approved treatment for this condition [2]. However, due to CSID’s nonspecific symptoms diagnosis is usually delayed. Prevalence of CSID may also be underrecognized due to overlapping presentation with other gastrointestinal (GI) conditions such as irritable bowel syndrome (IBS) and celiac disease, so the condition may be of greater clinical significance than previously suspected [3]. We present the case of a 50-year-old woman who presented with chronic intermittent diarrhea and bloating and was found to have CSID after an extensive GI workup including endoscopy, blood test, stool test and treatment of small intestinal bacterial overgrowth.

## Case presentation

**Figure 1:**
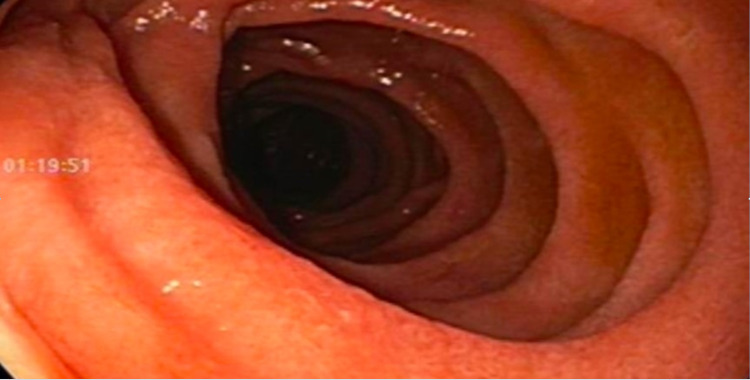
Upper endoscopy of 3rd portion duodenum with normal duodenal mucosa

A 50-year-old Caucasian female with past medical history of hypothyroidism was referred to the GI clinic for complaints of gradually worsening symptoms of intermittent bloating, non-bloody diarrhea (3-4 times a week), flatulence and burping for the last six months. The patient was unable to provide a clear relationship of her symptoms to any specific type of food and denied any symptoms when she is sleeping. She denied any overt bleeding, unintentional weight loss, and significant family history of gastrointestinal malignancies, celiac disease or inflammatory bowel disease. In addition to levothyroxine, the patient was occasionally using ibuprofen (3-4 times a week) for knee pain. She tried over-the-counter omeprazole for two months without any relief of symptoms. Patient’s physical examination was completely unremarkable. Her laboratory tests including thyroid function tests, C-reactive protein, IgA TTG, total IgA level and stool studies were unremarkable. Stool studies included fecal calprotectin, bacterial and viral cultures, C-diff PCR, and ova and parasites. She was advised to discontinue ibuprofen use. An upper endoscopy was performed which came back normal (Figure 1).

**Figure 2:**
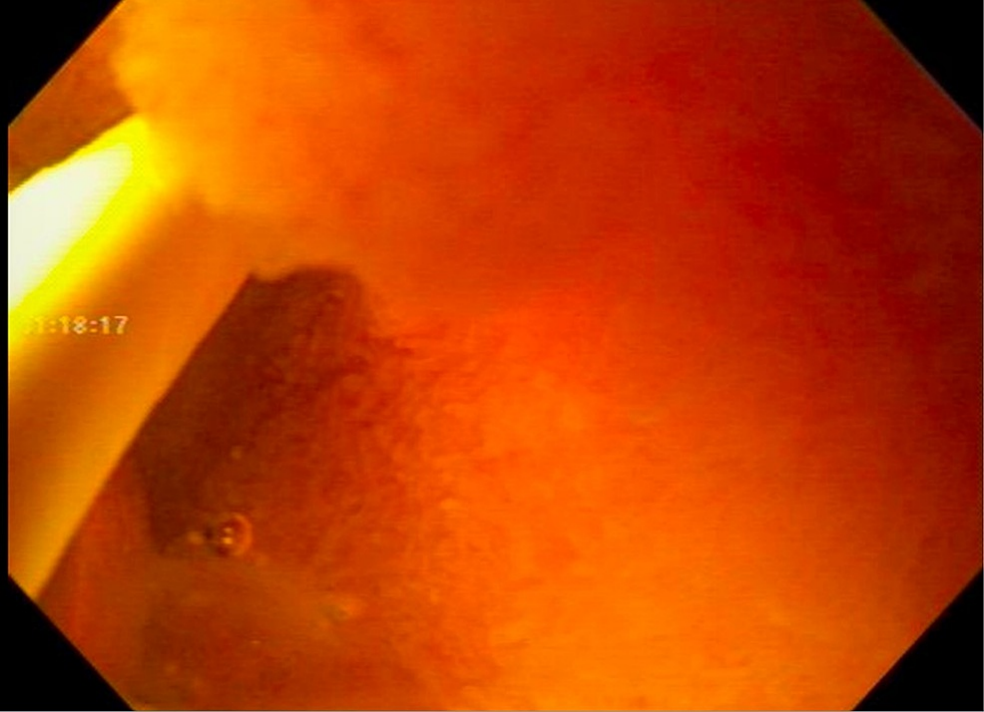
Duodenal aspirates to evaluate for small intestinal bacterial and fungal overgrowth

Duodenal biopsies came back with no evidence of celiac disease. Random gastric biopsies came back negative for H. pylori. Her colonoscopy with random colon biopsies was also negative for any evidence of microscopic colitis and inflammatory bowel disease (IBD). Subsequently, a glucose hydrogen breath test was positive for small intestinal bacterial overgrowth (SIBO). She was treated with Rifaximin 550mg TID x 14 days twice with only partial relief of symptoms. Due to persistent symptoms, she underwent repeat upper endoscopy with planned duodenal aspirates to evaluate and direct treatment of SIBO and small intestinal fungal overgrowth (SIFO) via culture and sensitivity as well as small bowel biopsies to evaluate disaccharidase deficiency assay (Figure 2). Duodenal aspirates were obtained by placing a 6 French liguory catheter into the channel of the gastroscope into the 3rd portion of the duodenum under aseptic precautions, including wearing sterile gloves by nurse and physician. Approximately 3 cc of bile-tinged duodenal fluid were aspirated and sent to microbiology for aerobic, anaerobic, and fungal cultures.

Her duodenal aspirate cultures were negative for SIBO and SIFO. However, her disaccharidase assay results were consistent with sucrase-isomaltase deficiency with sucrase, palatinase, and maltase values markedly low (Table 1).

**Table 1:** Disaccharidase assay and interpretation Interpretation: consistent with a sucrase-isomaltase deficiency

Enzyme	Value (uM/min/g protein)	Reference range
Lactase	43.0	>15.0
Sucrase	0.0	>25.0
Maltase	26.1	>100.0
Palatinase	0.0	>5.0

The patient was then referred to a dietician and started on sacrosidase enzyme supplement with complete resolution of symptoms.

## Discussion

The first reported case of CSID in 1960 was discovered in children with nonspecific gastrointestinal symptoms of osmotic diarrhea, steatorrhea, and vomiting after consumption of sucrose. In an effort to prevent these symptoms, adequate nutrition from sucrose-containing foods was not received, resulting in failure to thrive, and dehydration [4]. CSID is a rare autosomal inherited inborn error of metabolism that affects males and females in equal numbers. The condition is present in 0.2-0.5% of individuals of North American and European descent, but has a much higher prevalence in native populations of Greenland, Canada, and Alaska, ranging from approximately 3-10% [5-7]. So far, 27 mutations have been identified and linked to CSID, with the four most commonly involved mutations being G1073D, F1745C, V577G, and R1124X. Due to the relatively large number of genetic variations discovered in CSID, phenotypic variations with associated ranges in symptoms exist (compound heterozygosity). For example, all individuals with CSID have reduced sucrase activity, but some individuals may have normal isomaltase function while others have only trace isomaltase activity [8]. Heterozygous carriers of certain CSID mutations may also experience the nonspecific gastrointestinal symptoms associated with CSID in homozygous individuals [9]. Due to the numerous mutations in the sucrase-isomaltase genes responsible for CSID phenotypes, some individuals with CSID may present with homozygosity, while others may inherit compound heterozygosity [10, 11].

Sucrase-isomaltase (SI) is an enzyme found in the microvilli brush border of the small intestine and assists in the breakdown of disaccharidases like sucrose and products of starch digestion such as dextrins. Normally in a cell, SI is folded, N- and O-glycosylated in the medial Golgi and follows the secretory pathway to be placed on the apical cell surfaces of the brush border [12]. Along the secretory pathway, wild type SI dimerizes in medial Golgi cisternae; monomers interact via transmembrane domains like the “kin recognition” model of medial Golgi enzymes [13, 14]. Mutations in SI may involve altered folding, aberrant trafficking, missorting, and functional deficits in the normally folded enzyme and can be divided into heterogeneous classes based on function and secretory transport, possibly resulting in intracellular accumulation [13].

While primary, congenital SI deficiency is inherited, acquired forms of SI deficiency may be secondary to other chronic gastrointestinal conditions associated with intestinal villous atrophy, such as enteric H. pylori infection, celiac disease, Crohn’s disease, and other enteropathies affecting the small intestine [15, 16]. To further complicate detection and diagnosis of CSID, the predominant symptoms of CSID have been found to overlap with common presentations of irritable bowel syndrome (IBS). Furthermore, CSID and IBS both have heterogeneous phenotypic manifestations due to their variability in the trafficking and function of associated mutant enzymes [17]. Although genetic testing for SI mutations in individuals presenting with IBS-like symptoms can be done to confirm congenital nature of SI deficiency, it is not routinely done in practice and can be highly costly. It is rather important to acquire endoscopic, biopsy, and culture studies for any abnormalities or structural changes to rule out secondary causes of SI deficiency.

The similarity of symptom manifestations between chronic GI disorders and SI deficiency adds to the diagnostic dilemma that clinicians face. Since CSID is believed to be rare, and symptoms of starch intolerance generally resolve in childhood, a high index of suspicion is required to even consider the diagnosis, the prevalence of CSID maybe higher, under recognized and undertreated in the adult population [17, 18].

There are many diagnostic tools that may be used to diagnose CSID. Regarded as gold standard for diagnosis, the disaccharidase assay is an invasive test that utilizes small bowel biopsy to determine functional enzyme activity levels. The assay shows the levels of various enzymes such as sucrase-isomaltase (SI), lactase, and maltase. Results are consistent with CSID if the amount of sucrose broken down by SI is lower than expected [19]. The invasiveness of obtaining these biopsies for disaccharidase assays led to the development of non-invasive tests that investigate gas production from enzymatic activity. There are two commercially available non-invasive sucrose breath test in the market. The carbon-13 (13C) sucrose breath involves intake of a sucrose with a stable version of carbon known as carbon-13. If there is a deficiency in functional SI enzymes in the intestine, then the amount of carbon-13 gas exhaled will be reduced. This test is regarded as the most direct and definitive measure in detecting CSID [19]. Additionally, the sucrose hydrogen-methane breath test measures levels of hydrogen gas exhaled after consumption of a sucrose solution. In the absence of intestinal SI, the amount of hydrogen gas exhaled will be increased. This breath test is more indirect, less specific, and less accurate in diagnosing CSID compared to the carbon-13 breath test due to other reasons for elevated hydrogen gas in exhaled breath [19, 20].

The only effective and FDA-approved drug treatment option for CSID is enzyme replacement therapy with sacrosidase [2]. The drug is only available with prescription but may be challenging to obtain due to unavailability at local pharmacies. Sacrosidase prescriptions must be filled through a specialty pharmacy. While the drug is a treatment for CSID, it is not a cure. Thus, treatment is usually a combination of dietary modifications, such as limiting high sucrose and starch-containing foods, with the assistance of a registered dietician well versed with CSID and enzyme replacement therapy.

## Conclusions

We present the case of a woman with complaints of chronic persistent bloating, diarrhea, flatulence, and burping despite undergoing an extensive GI diagnostic evaluation including esophagogastroduodenoscopy (EGD), colonoscopy with mucosal biopsies, treatment of SIBO with partial relief of symptoms before being diagnosed with CSID through a positive disaccharidase assay. This case highlights the presence of CSID in an adult with symptoms like those of a chronic GI disease. Given the overlapping presentations of CSID and IBS, it is important for physicians to consider screening for certain gastrointestinal complaints using non-invasive and invasive tools such as breath tests and biopsy with disaccharidase assay, respectively, to diagnose SI deficiency. Specific management approaches for SI deficiency differs significantly from other chronic conditions given the availability of sacrosidase, which currently is the only FDA-proven enzyme-replacement therapy for symptom relief. The possibility of underestimating the prevalence of CSID in the adult population especially with chronic persistent non-specific GI symptoms should be further investigated.
